# The status quo of systematic reviews published in high-impact journals in Korea: a study focused on protocol registration and GRADE use

**DOI:** 10.4178/epih.e2022108

**Published:** 2022-11-15

**Authors:** Mi Ah Han, Seong Jung Kim, Eu Chang Hwang, Jae Hung Jung

**Affiliations:** 1 Department of Preventive Medicine, Chosun University College of Medicine, Gwangju, Korea; 2 Department of Internal Medicine, Chosun University College of Medicine, Gwangju, Korea; 3 Department of Urology, Chonnam National University Medical School, Chonnam National University Hwasun Hospital, Hwasun, Korea; 4 Department of Urology, Yonsei University Wonju College of Medicine, Wonju, Korea; 5 Center of Evidence Based Medicine, Institute of Convergence Science, Yonsei University, Seoul, Korea

**Keywords:** Editorial policies, GRADE approach, Guidelines. Journal article, Research report, Systematic review

## Abstract

**OBJECTIVES:**

This study investigated the status quo of systematic reviews published in major journals in Korea from the perspective of protocol registration and adopting the grading of recommendation, assessment, development and evaluation (GRADE) system.

**METHODS:**

We examined systematic reviews published in Korea’s top 15 medical journals from 2018 to 2021. Teams of 2 reviewers assessed the studies’ eligibility criteria and extracted data independently and in duplicate. We collected information on study characteristics, protocol registration, and GRADE use of the included reviews, and reviewed the “Instructions for Authors” of the selected journals to assess any guidance related to systematic reviews.

**RESULTS:**

Out of the 126 identified reviews, 18 (14.3%) reported that they registered or published their protocol. Only 5 (4.0%) rated the certainty of evidence; and all 5 used the GRADE system. Only 6 of 15 journals mentioned systematic reviews in their “Instructions for Authors.” Six journals endorsed the Preferred Reporting Items for Systematic Reviews and Meta-Analyses (PRISMA) framework for systematic review reporting (2 mandatory, 3 recommended, and 1 unclear). None of the journals included mentioned protocol registration or certainty of evidence in their authors’ guidelines.

**CONCLUSIONS:**

Overall, the proportion of systematic reviews that had prior protocol registration or used the GRADE approach to rate the certainty of evidence was very low. Our study highlights the need for adherence to systematic review standards in medical journals in Korea, including prior protocol registration and certainty of evidence assessment. Our review will help improve the quality of systematic reviews in Korea.

## GRAPHICAL ABSTRACT

[Fig f1-epih-44-e2022108]

## INTRODUCTION

Systematic reviews could provide the best insights into the state of a field by summarizing the available evidence when conducted with adherence to rigorous standards. With the growing importance of systematic reviews in clinical and public health, methodological guidelines have addressed important issues about the standards for conducting systematic reviews.

One of the issues is protocol registration before conducting a systematic review. Prospectively registering the protocol on free public-access websites such as the International Prospective Register of Systematic Reviews (PROSPERO) or publishing it in a journal has many benefits such as preventing duplicated research and selective reporting of findings, promoting completeness of research, improving the transparency and accuracy of the research process, and enhancing the reliability of the findings [1]. Therefore, the Preferred Reporting Items for Systematic Reviews and Meta-Analyses (PRISMA), a reporting guideline for systematic reviews, considers protocol registration an important reporting item [2].

The grading of recommendation, assessment, development and evaluation (GRADE) system is a tool to evaluate the certainty of evidence and assess the overall evidence by considering factors that decrease the quality of evidence (e.g., study design and inconsistency) and those that increase it (e.g., a dose-response relationship) [3].

Recently, we evaluated the prevalence and methodological quality of systematic reviews published in medical journals in Korea [4]. We inferred that the methodological quality of the 126 systematic reviews published in the last 4 years from 2018 to 2021 was low to very low according to A MeaSurement Tool to Assess Systematic Reviews 2 (AMSTAR 2), a quality assessment tool for systematic reviews [5]. AMSTAR 2 consists of a total of 16 items (7 critical and 9 non-critical), explicitly considers protocol registration as a critical aspect of high-quality systematic reviews, and regards the domains of the certainty of evidence assessment (such as risk of bias, inconsistency, and publication bias) as critical domains for high-quality systematic reviews. Despite the endorsement of methodological guidelines and international trends, we found that prospective protocol registration was insufficient and the certainty of evidence reporting was limited.

Although these findings could indicate the authors’ ignorance, they could also indicate the need for training for journal editors and reviewers. The editorial policies of a journal reflect its scope and publishing standards. The authors are expected to adhere to these policies to publish their studies. Therefore, journal editorial policies could affect studies’ methodological standards and reporting quality. Statements describing the editorial policies of journals are typically found in the “Instructions for Authors,” indicating requirements or guidelines for authors to adhere to when submitting a manuscript to be considered for publication.

This study aimed to present the status of systematic reviews published in major Korean journals, focusing on protocol registration and the use of GRADE, based on our previous review. We additionally reviewed the author guidelines of the journals included in the review to identify guidelines related to systematic reviews.

## MATERIALS AND METHODS

We used previously collected data to investigate the methodological quality of systematic reviews in medical journals in Korea. The review protocol was registered (CRD42021283214), a full description of the study’s review methods and findings were made available [4]. The primary study process is presented below.

### Search and inclusion criteria

We searched major medical journals on the KoreaMed website (http://koreamed.org), a service provided by the Korean Association of Medical Journal Editors on October 18, 2021. We selected the top 15 journals with the highest Korean Medical Citation Index (KoMCI)—a measure of citations within a database maintained by the Korean Academy of Medical Science: *Allergy Asthma Immunol Res, Asian Oncol Nurs, Cancer Res Treat, Clin Psychopharmacol Neurosci, Diabetes Metab J, J Bone Metab, J Korean Acad Nurs, J Korean Acad Nurs Adm, J Korean Med Sci, J Korean Soc Clin Toxicol, J Menopausal Med, J Stroke, Korean J Radiol, Perspect Nurs Sci, and Ultrasonography*.

We reviewed all publications of these 15 journals from 2018 to 2021, since AMSTAR-2 was published in 2017, and identified all systematic reviews. We regarded a study as a systematic review when authors explicitly reported that they conducted a systematic review using terms such as “systematic” or “systematically.” Moreover, we considered studies as systematic reviews when authors described some steps of systematic review standards such as study searches and study selection processes that could be reproducible.

### Study selection and data extraction

Teams of 2 reviewers each performed reference screening and data extraction. At each stage, we conducted a calibration exercise that was documented to ensure that the reviewers understood the study objective and inclusion criteria in detail.

We used pre-piloted data extraction forms and collected study characteristics and GRADE use from the systematic reviews. Additionally, we reviewed the author’s guidelines, “Instructions for Authors” of each journal, and whether the journal recommended or mandated protocol registration.

#### The study characteristics of the systematic reviews comprised the following details

First author, international collaborative authorship (no, yes), publication year, language of publication (Korean or English), type of question (intervention/therapy or others), study design of primary studies (randomized controlled trials [RCTs], non-RCTs, or both), number of primary studies, number of participants, type of intervention or exposure, type of outcome, meta-analysis (yes or no), whether PRISMA guidelines were followed (yes or no), and whether the overall certainty of evidence was rated (yes or no).

The journal characteristics from the “Instructions for Authors” of the journals comprised the following details

Language of publication (Korean only, English only, or both), Science Citation Index (Expanded) listing (yes or no), whether systematic reviews were mentioned in the author guidelines (yes or no), endorsement or enforcement of reporting guidelines of a systematic review article (yes or no, if yes, the name of the reporting guideline), protocol registration (yes or no), and rating the certainty of evidence (yes or no).

For each of the abovementioned guidelines, the level of endorsement or enforcement was documented as required or mandatory (an essential criterion for manuscript acceptance), recommended (usage encouraged, but not mandatory), and unclear (guidelines were mentioned, but the necessity of their inclusion in the manuscript remained ambiguous).

### Statistical analysis

We presented the general characteristics of the journals with descriptive statistics. Furthermore, we presented the proportion of protocol registration or publication of systematic reviews and compared them to the characteristics of the studies using the chi-square test. Subsequently, we provided descriptions of the journals’ guidelines regarding systematic reviews. All statistical analyses were performed using SAS version 9.4 (SAS Institute Inc., Cary, NC, USA).

### Ethics statement

Ethics approval is not required because we only used data from published papers and journals’ websites.

## RESULTS

### Protocol registration of systematic reviews

Only 14.3% (18 of 126) of systematic reviews registered their protocol. However, the proportion of registration increased from 6.7% in 2018 to 33.3% in 2021. Reviews with international collaborative authorship exhibited a higher registration rate than those by Korean authors (Table 1). Supplementary Material 1 presents the detailed characteristics of the 126 systematic reviews included.

### Grading of recommendation, assessment, development and evaluation adoption by systematic reviews

Five studies [6-10] rated the certainty of evidence, and all 5 reviews used the GRADE system to do so. All of these studies were published in English, and 4 studies were interested in intervention questions. The number of primary studies ranged from 11 to 30, and 4 reviews conducted meta-analyses; they reported that they adhered to reporting guidelines. None of these studies registered their protocol (Table 2).

### Author guidelines of the journals

We found that the author guidelines of 6 of the 15 journals mentioned systematic reviews. Six journals mentioned PRISMA as their reporting guidelines for systematic reviews; however, the level of endorsement or enforcement was required or mandatory in 2, recommended in 3, and unclear in 1. No instructions mentioned protocol registration and certainty of evidence assessment in the main body of the authors’ guidelines (Table 3). Supplementary Material 2 presents the detailed characteristics of 15 journals included.

## DISCUSSION

### Main findings

We found that 14.3% of systematic reviews registered their protocol and only 5 studies used the GRADE method to rate the certainty of evidence. In addition, the authors’ guidelines in the journals rarely provided specific guidelines for systematic reviews.

### Strength and limitations

Our study primarily addresses the status quo of protocol registration and GRADE use in systematic reviews in major medical journals in Korea. Our study adhered to the standards of this area, including duplicate and independent reference screening and data extraction, in addition to calibration exercises to ensure accuracy and reliability.

This study has limitations. First, we evaluated protocol registration based on the studies’ reports and did not check the open-access online database of systematic review protocols such as PROSPERO. However, we believe that it would be unlikely for papers to register their protocol but not mention it. Second, due to the low number of studies that registered their protocol and used the GRADE system, we could not conduct a multivariable analysis to identify factors related to protocol registration or a comparative analysis to investigate study characteristics associated with GRADE use. Third, since this study identified the characteristics of journals from only 15 journals that published systematic reviews included based on a quality assessment, we could not provide comprehensive information on trends in the policies or characteristics of Korean journals related to systematic reviews.

### Relation to previous works

Several studies have investigated the protocol registration of systematic reviews. On one such study, out of 284 included reviews, 60 (21%) protocols were registered. The proportion of registration increased from 5.6% in 2009 to 27% in 2015 (p for trend <0.001) [11]. Out of 495 systematic reviews in dentistry, 162 (32.7%) reported registering the systematic review protocol or working from a previously established protocol; moreover, it must be noted that previously registering a protocol positively influenced the final report quality of systematic reviews in dentistry [12]. Although the registration proportion of reviews in this study was low compared to previous studies, it appears to have increased from 2018 to 2021. In a survey of global researchers, 44.2% did not register their protocols prior to publishing their systematic reviews, and the main reasons for not registering their protocol were lack of knowledge about protocol registration (44.9%), it was not mandatory (43.0%), and the benefits of registration were unknown (35.0%) [13]. Therefore, there is a need to make prior protocol registration mandatory, as well as raising researchers’ awareness of the benefits of protocol registration.

Only 5 reviews (4.0% of 126) used GRADE to rate the certainty of evidence in this study. In previous studies, the proportions of GRADE use in systematic reviews substantially varied depending on the research area or publishing institution. For example, out of 800 systematic reviews in high-impact-factor nutrition journals, 55 (6.9%) used GRADE to rate the certainty of evidence, with a slight increase in the GRADE use by publication year [14]. Among Cochrane systematic reviews of traditional Chinese medicine, 86 (38.1%) used the GRADE approach to rate the certainty of evidence [15]. In the included systematic review, the authors presented the distribution of domains determining the certainty of evidence, such as the risk of bias and imprecision, and emphasized the wider adoption of GRADE for transparent and evidence-based decision-making. Due to the limited number of studies using GRADE in our review, we could not reveal any trends and characteristics related to GRADE use; therefore, we recommend finding support for our findings using a larger sample.

Previous studies have investigated the authors’ guidelines of journals to examine the journals’ policies on systematic reviews. In a sample of 146 journals that published systematic reviews indexed in the 2009 Journal Citation Index, the PRISMA statement was referred to in the instructions for authors in 27% (40 of 146) of journals, 50% in general and internal medicine journals (7 of 14), and 25% in specialty medicine journals (33 of 132) [16]. Among 134 surgery journals indexed in the 2009 Journal Citation Report, the Instructions for Authors of only 1 journal mentioned registration for systematic reviews [17]. Out of 80 dermatology journals, 38.8% mentioned systematic reviews in their author guidelines, while only 2 (2.5%) required PROSPERO registration in their author guidelines [18]. Aligning with previous studies, journals did not provide guidelines for systematic review authors or endorsement of relevant guidelines to encourage improving in reporting and registration.

### Implications

Most reviews did not register their proposals prospectively, and journals did not provide relevant guidelines for the authors. Authors should be aware that prospective registration of the protocol is necessary for the transparency and credibility of their review. In addition, journals and editors should specify guidelines that reflect the latest high-quality standard guidelines for systematic reviews, inform authors, and educate reviewers accordingly.

Systematic reviews have been recognized as tools that can systematically appraise and summarize the available evidence on a health issue. Assessing the certainty of evidence acknowledges the methodological limitations of the evidence found and provides an accurate view of how confident we are in it. The GRADE approach is the best tool available to assess the certainty of evidence and facilitates adherence to methodological standards, comparison of results between studies, and application of results to the field.

In conclusion, notifying authors, reviewers, and editors of the importance of prospective protocol registration and evaluating the certainty of evidence using GRADE could improve the transparency and credibility of systematic reviews. Based on this, our study presented the status quo of Korean journals and found that the prospective registration of systematic review protocols was low and that GRADE use was very low in medical journals in Korea. Our results could be used to revise journal guidelines, educate authors, reviewers, and editors, and ultimately improve the quality of systematic reviews in Korea.

## Figures and Tables

**Figure f1-epih-44-e2022108:**
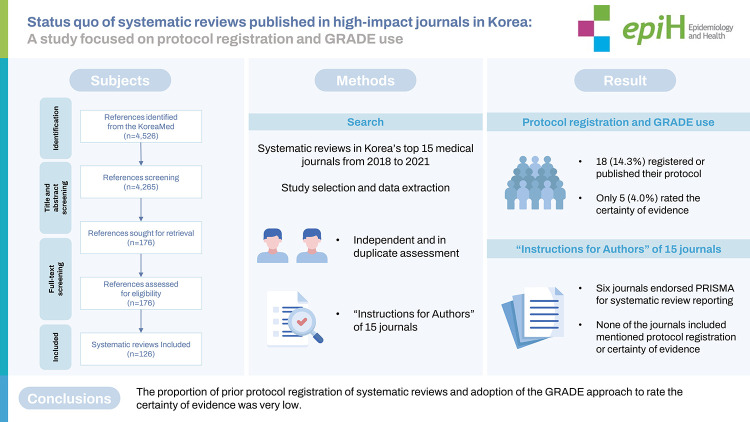


**Table 1.** Characteristics of included studies according to protocol registration

Characteristics		Total	Protocol registration or publication		p-value
			No	Yes	
Total		126 (100)	108 (85.7)	18 (14.3)	
Publication year					0.007
	2018	30 (23.8)	28 (93.3)	2 (6.7)	
	2019	29 (23.0)	27 (93.1)	2 (6.9)	
	2020	37 (29.4)	33 (89.2)	4 (10.8)	
	2021	30 (23.8)	20 (66.7)	10 (33.3)	
International collaborative authorship					0.036
	Korea only	69 (54.8)	64 (92.8)	5 (7.3)	
	Other countries only	50 (39.7)	38 (76.0)	12 (24.0)	
	Korea and other countries	7 (5.6)	6 (85.7)	1 (14.3)	
Language of publication					0.711
	English	102 (81.0)	88 (86.3)	14 (13.7)	
	Korean	24 (19.1)	20 (83.3)	4 (16.7)	
Type of question					0.102
	Intervention	76 (60.3)	62 (81.6)	14 (18.4)	
	Other	50 (39.7)	46 (92.0)	4 (8.0)	
Type of included studies					0.389
	Observational studies only	52 (41.3)	46 (88.5)	6 (11.5)	
	Randomized controlled trials only	45 (35.7)	36 (80.0)	9 (20.0)	
	Both	29 (23.0)	26 (89.7)	3 (10.3)	
Total no. of primary studies included					0.307
	≤10	34 (27.0)	31 (91.2)	3 (8.8)	
	11-20	42 (33.3)	37 (88.1)	5 (11.9)	
	≥21	50 (39.7)	40 (80.0)	10 (20.0)	
Total no. of participants included					0.362
	≤1,000	38 (30.2)	34 (89.5)	4 (10.5)	
	1,001-5,000	42 (33.3)	36 (85.7)	6 (14.3)	
	≥5,001	38 (30.2)	30 (79.0)	8 (21.1)	
	NR	8 (6.4)	8 (100)	0 (0.0)	
Type of intervention/exposure					0.142
	Therapeutic clinical intervention	71 (56.4)	58 (81.7)	13 (18.3)	
	Others	55 (43.7)	50 (90.9)	5 (9.1)	
Type of outcome					0.031
	Morbidity	51 (40.5)	44 (86.3)	7 (13.7)	
	Symptoms	20 (15.9)	14 (70.0)	6 (30.0)	
	Mortality	28 (22.2)	23 (82.1)	5 (17.9)	
	Others	27 (21.4)	27 (100)	0 (0.0)	
Meta-analysis					0.049
	No	30 (23.8)	29 (96.7)	1 (3.3)	
	Yes	96 (76.2)	79 (82.3)	17 (17.7)	
Adherence to reporting guideline (e.g., PRISMA)					0.358
	No	32 (25.4)	29 (90.6)	3 (9.4)	
	Yes	94 (74.6)	79 (84.0)	15 (16.0)	

Values are presented as number (%). NR, not reported; PRISMA, Preferred Reporting Items for Systematic Reviews and Meta-Analyses.

**Table 2.** Characteristics of studies with GRADE use

Characteristics	Hsu 2018 [6]	Park 2018 [7]	Kulthanan 2019 [8]	Ashe 2021 [9]	Kim 2021 [10]
Publication year	2018	2018	2019	2021	2021
International collaborative authorship	Other countries only	Korea only	Other countries only	Other countries only	Korea & other countries
Language of publication	English	English	English	English	English
Type of question	Intervention	Intervention	Prognosis	Intervention	Intervention
Type of included studies	RCTs only	Both	Both	RCTs only	Both
Total no. of primary studies included	13	30	13	11	11
Total no. of participants included	65,812	NR	604	723	NR
Type of intervention/exposure	Therapeutic clinical intervention	Therapeutic clinical intervention	Others	Health behavior	Therapeutic clinical intervention
Type of outcome	Morbidity	Symptoms	Others	Morbidity	Biophysical status
Meta-analysis	Yes	Yes	No	Yes	Yes
Adherence to reporting guideline (e.g., PRISMA)	Yes	Yes	Yes	Yes	Yes
Protocol registration or publication	No	No	No	No	No

GRADE, grading of recommendation, assessment, development and evaluation; NR, not reported; RCT, randomized controlled trial; PRISMA, Preferred Reporting Items for Systematic Reviews and Meta-Analyses.

**Table 3.** Authors’ guidelines of the journals included

Variables	n (%)
Publication language	15 (100)
Korean only	5 (33.3)
English only	5 (33.3)
Both	5 (33.3)
SCI(E)	
No	6 (40.0)
Yes	9 (60.0)
Mentioning systematic reviews	
No	9 (60.0)
Yes	6 (40.0)
Adherence to reporting guideline	
No description	9 (60.0)
Yes, with PRISMA	6 (40.0)
Level of endorsement for reporting guideline	
Required/mandatory	2 (13.3)
Recommended	3 (20.0)
Unclear	1 (6.7)
Not applicable	9 (60.0)
Protocol of systematic reviews	
No description	15 (100)
Recommended	0 (0.0)
Certainty of evidence	
No description	15 (100)
Recommended	0 (0.0)

SCI(E), Science Citation Index (Expanded); PRISMA, Preferred Reporting Items for Systematic Reviews and Meta-Analyses.

## SUPPLEMENTARY MATERIALS

Supplementary Material 1. General characteristics of systematic reviews included

Supplementary Material 2. Author guidelines of journals included
