# Supplementary material for: The status quo of systematic reviews published in high-impact journals in Korea: a study focused on protocol registration and GRADE use
Source: Epidemiol Health. 2022 Nov 15;44:e2022108. doi: 10.4178/epih.e2022108 (PMC10185969; doi:10.4178/epih.e2022108)
Supplement: Supplementary Material 1. — General characteristics of systematic reviews included [file epih-44-e2022108-Supplementary-1.docx]

Supplementary Material 1. General characteristics of systematic reviews included

| First author, publication year | Language of publication | Type of question | Type of included studies | Total number of primary studies included | Total number of participants included | Primary intervention/exposure | Primary outcome | Meta-analysis |
| --- | --- | --- | --- | --- | --- | --- | --- | --- |
| Al-Hader 2019 | English | Prevalence | OS | 187 | 184922 | Therapeutic clinical intervention | Morbidity | conventional MA |
| AlHajri 2021 | English | Intervention | RCT | 9 | 1285 | Therapeutic clinical intervention | Biophysical status | no MA conducted |
| Aliena-Valero 2021 | English | Diagnosis | OS | 134 | 6661 | Diagnostic test | Morbidity | no MA conducted |
| Ashe 2021 | English | Intervention | RCT | 11 | 723 | Health behavior | Morbidity | conventional MA |
| Babakhanian 2018 | English | Intervention | RCT | 4 | 296 | Therapeutic clinical intervention | Symptoms | conventional MA |
| Bae 2020 | Korean | Intervention | Both | 9 | 5900 | Therapeutic clinical intervention | Symptoms | conventional MA |
| Becic 2018 | English | Intervention | RCT | 14 | 685 | Therapeutic clinical intervention | Biophysical status | conventional MA |
| Bejleri 2021 | English | Diagnosis | OS | 22 | 4190 | Diagnostic test | Morbidity | no MA conducted |
| Bhatia 2020 | English | Prognosis | OS | 30 | 115 | Biological status | Mortality | no MA conducted |
| Cai 2021 | English | Intervention | RCT | 57 | 13240 | Therapeutic clinical intervention | Biophysical status | conventional MA |
| Cha 2020 | English | Intervention | RCT | 8 | 822 | Therapeutic clinical intervention | Morbidity | network MA |
| Cha 2020 | English | Other | Both | 65 | 45942 | Others | Others | no MA conducted |
| Chae 2021 | Korean | Intervention | RCT | 17 | 1494 | Therapeutic clinical intervention | Others | conventional MA |
| Chang 2018 | English | Intervention | RCT | 41 | 1972 | Therapeutic clinical intervention | Mortality | conventional MA |
| Chang 2021 | English | Intervention | RCT | 16 | 897 | Therapeutic clinical intervention | Symptoms | conventional MA |
| Cho 2020 | English | Intervention | Both | 17 | 999 | Therapeutic clinical intervention | Others | conventional MA |
| Choi 2018 | English | Intervention | OS | 13 | 567 | Therapeutic clinical intervention | Mortality | conventional MA |
| Choi 2018 | English | Intervention | OS | 60 | 9428076 | Health behavior | Morbidity | conventional MA |
| Choi 2018 | Korean | Intervention | OS | 13 | 5125 | Therapeutic clinical intervention | Mortality | no MA conducted |
| Choi 2020 | English | Intervention | OS | 14 | 495 | Therapeutic clinical intervention | Mortality | conventional MA |
| Choi 2020 | Korean | Intervention | RCT | 11 | 1153 | Therapeutic clinical intervention | Symptoms | conventional MA |
| Chung 2019 | Korean | Intervention | OS | 23 | 24 | Therapeutic clinical intervention | Others | no MA conducted |
| Chung 2019 | English | Diagnosis | OS | 12 | 418 | Diagnostic test | Morbidity | no MA conducted |
| Chung 2019 | Korean | Other | OS | 36 | 12154 | Others | Others | conventional MA |
| DeCagna 2019 | English | Intervention | RCT | 15 | 381 | Therapeutic clinical intervention | Morbidity | no MA conducted |
| Fusar-Poli 2021 | English | Prognosis | OS | 43 | 9484 | Biological status | Morbidity | conventional MA |
| Ha 2020 | Korean | Intervention | OS | 7 | 1017 | Therapeutic clinical intervention | Morbidity | conventional MA |
| Heidari 2019 | English | Prognosis | OS | 27 | 28088 | Biological status | Biophysical status | no MA conducted |
| Hong 2019 | English | Intervention | RCT | 6 | 249 | Therapeutic clinical intervention | Biophysical status | conventional MA |
| Hsu 2018 | English | Intervention | RCT | 13 | 65812 | Therapeutic clinical intervention | Morbidity | conventional MA |
| Hwang 2018 | Korean | Intervention | Both | 27 | 3110 | Health behavior | Others | no MA conducted |
| Hwang 2020 | Korean | Intervention | Both | 32 | 1663 | Others | Others | no MA conducted |
| Igwe 2018 | English | Intervention | RCT | 5 | 183 | Therapeutic clinical intervention | Morbidity | conventional MA |
| Jang 2019 | English | Intervention | RCT | 23 | 723 | Health behavior | Biophysical status | conventional MA |
| Jeon 2018 | English | Prognosis | OS | 8 | 21930 | Biological status | Morbidity | conventional MA |
| Jung 2018 | Korean | Intervention | RCT | 8 | 855 | Therapeutic clinical intervention | Symptoms | conventional MA |
| Kang 2019 | English | Intervention | Both | 17 | 17511 | Therapeutic clinical intervention | Mortality | conventional MA |
| Kang 2020 | English | Prevalence | Both | 45 | 6127 | Others | Others | no MA conducted |
| Kerleroux 2021 | English | Intervention | RCT | 10 | 1941 | Therapeutic clinical intervention | Symptoms | conventional MA |
| Khadivzadeh 2018 | English | Intervention | RCT | 3 | 225 | Therapeutic clinical intervention | Others | conventional MA |
| Khadivzadeh 2018 | English | Intervention | RCT | 10 | 469 | Therapeutic clinical intervention | Symptoms | conventional MA |
| Khadivzadeh 2018 | English | Intervention | RCT | 12 | 1257 | Therapeutic clinical intervention | Others | conventional MA |
| Khaleghi 2020 | English | Intervention | Both | 32 | 609 | Therapeutic clinical intervention | Symptoms | no MA conducted |
| Khanra 2021 | English | Intervention | Both | 10 | 321844 | Therapeutic clinical intervention | Morbidity | network MA |
| Kim 2019 | Korean | Intervention | RCT | 23 | 1527 | Therapeutic clinical intervention | Symptoms | conventional MA |
| Kim 2019 | English | Intervention | Both | 17 | 1321 | Therapeutic clinical intervention | Mortality | conventional MA |
| Kim 2019 | English | Other | OS | 516 | NR | Others | Others | no MA conducted |
| Kim 2019 | English | Diagnosis | OS | 34 | 6196 | Diagnostic test | Biophysical status | conventional MA |
| Kim 2019 | Korean | Intervention | RCT | 22 | 1461 | Therapeutic clinical intervention | Symptoms | conventional MA |
| Kim 2019 | English | Prognosis | OS | 20 | 47930 | Biological status | Morbidity | conventional MA |
| Kim 2020 | English | Diagnosis | OS | 20 | 766 | Diagnostic test | Others | conventional MA |
| Kim 2020 | English | Diagnosis | OS | 13 | 2136 | Diagnostic test | Morbidity | conventional MA |
| Kim 2021 | English | Prevalence | OS | 55 | 13527 | Others | Morbidity | conventional MA |
| Kim 2021 | English | Intervention | OS | 4 | 653 | Therapeutic clinical intervention | Morbidity | conventional MA |
| Kim 2021 | English | Intervention | Both | 11 | NR | Therapeutic clinical intervention | Biophysical status | conventional MA |
| Kim 2021 | English | Diagnosis | OS | 16 | 4000 | Diagnostic test | Biophysical status | conventional MA |
| Kim 2021 | Korean | Intervention | RCT | 37 | 3491 | Therapeutic clinical intervention | Morbidity | conventional MA |
| Ko 2021 | English | Diagnosis | OS | 20 | 44513 | Diagnostic test | Others | conventional MA |
| Kronenburg 2018 | English | Prevalence | OS | 17 | 434 | Others | Others | no MA conducted |
| Kulthanan 2019 | English | Prognosis | Both | 13 | 604 | Others | Others | no MA conducted |
| Lally 2019 | English | Other | OS | 15 | 1360 | Biological status | Biophysical status | no MA conducted |
| Lee 2018 | English | Intervention | RCT | 9 | 4034 | Therapeutic clinical intervention | Symptoms | network MA |
| Lee 2019 | English | Intervention | RCT | 9 | 750 | Therapeutic clinical intervention | Symptoms | conventional MA |
| Lee 2019 | English | Intervention | OS | 8 | 10533 | Therapeutic clinical intervention | Morbidity | no MA conducted |
| Lee 2019 | English | Prognosis | OS | 7 | 3813 | Others | Morbidity | conventional MA |
| Lee 2020 | English | Intervention | RCT | 30 | 117974 | Therapeutic clinical intervention | Morbidity | conventional MA |
| Lee 2020 | English | Intervention | RCT | 37 | 1813 | Health behavior | Biophysical status | conventional MA |
| Lee 2020 | English | Intervention | OS | 4 | 127722 | Therapeutic clinical intervention | Morbidity | conventional MA |
| Lee 2020 | English | Intervention | RCT | 10 | 2126 | Therapeutic clinical intervention | Morbidity | conventional MA |
| Lee 2020 | Korean | Other | OS | 30 | 7943 | Others | Others | conventional MA |
| Lee 2021 | English | Intervention | RCT | 13 | 322 | Therapeutic clinical intervention | Symptoms | conventional MA |
| Liao 2018 | English | Diagnosis | OS | 27 | NR | Diagnostic test | Morbidity | conventional MA |
| Lim 2020 | Korean | Other | Both | 146 | NR | Others | Others | conventional MA |
| Lim 2021 | English | Diagnosis | OS | 22 | 1732 | Diagnostic test | Morbidity | conventional MA |
| Lin 2021 | English | Diagnosis | OS | 7 | 417 | Diagnostic test | Morbidity | conventional MA |
| Malhotra 2019 | English | Prognosis | OS | 26 | 56513 | Biological status | Others | conventional MA |
| Matusevicius 2021 | English | Prognosis | OS | 25 | 7543 | Biological status | Morbidity | conventional MA |
| MohdTahir 2020 | English | Other | Both | 26 | 1234 | Others | Biophysical status | conventional MA |
| Moriwaki 2021 | English | Intervention | Both | 15 | 2581 | Therapeutic clinical intervention | Mortality | conventional MA |
| Nassar 2018 | English | Intervention | Both | 33 | 2714502 | Therapeutic clinical intervention | Morbidity | conventional MA |
| Oh 2018 | Korean | Intervention | Both | 22 | 954 | Therapeutic clinical intervention | Symptoms | conventional MA |
| Papadopoulos 2020 | English | Prognosis | OS | 31 | 25562 | Biological status | Morbidity | conventional MA |
| Park 2018 | English | Intervention | Both | 30 | NR | Therapeutic clinical intervention | Symptoms | conventional MA |
| Park 2018 | Korean | Other | OS | 15 | 4049 | Others | Others | conventional MA |
| Park 2018 | Korean | Other | Both | 12 | 1226 | Others | Others | no MA conducted |
| Park 2020 | Korean | Intervention | OS | 30 | 1168 | Therapeutic clinical intervention | Morbidity | conventional MA |
| Park 2021 | English | Intervention | OS | 19 | 2502 | Therapeutic clinical intervention | Morbidity | conventional MA |
| Park 2021 | English | Diagnosis | OS | 25 | 4867 | Diagnostic test | Morbidity | conventional MA |
| Rahmani 2018 | English | Intervention | RCT | 11 | 1052 | Therapeutic clinical intervention | Morbidity | conventional MA |
| RashidiFakari 2020 | English | Intervention | RCT | 12 | 814 | Therapeutic clinical intervention | Morbidity | no MA conducted |
| Riazi 2019 | English | Intervention | RCT | 6 | 391 | Therapeutic clinical intervention | Biophysical status | no MA conducted |
| Rocha 2020 | English | Intervention | RCT | 9 | 394 | Therapeutic clinical intervention | Biophysical status | conventional MA |
| Romoli 2020 | English | Intervention | Both | 18 | 7017 | Therapeutic clinical intervention | Mortality | conventional MA |
| Roozbeh 2019 | English | Intervention | RCT | 4 | 310 | Therapeutic clinical intervention | Others | no MA conducted |
| Ryu 2018 | Korean | Intervention | RCT | 5 | 931 | Therapeutic clinical intervention | Others | conventional MA |
| Seo 2019 | Korean | Intervention | RCT | 21 | NR | Therapeutic clinical intervention | Symptoms | conventional MA |
| Seo 2021 | Korean | Intervention | RCT | 12 | 103855 | Therapeutic clinical intervention | Morbidity | conventional MA |
| Shim 2018 | English | Intervention | RCT | 11 | 2522 | Therapeutic clinical intervention | Symptoms | no MA conducted |
| Shin 2020 | English | Intervention | Both | 10 | 3229 | Therapeutic clinical intervention | Morbidity | no MA conducted |
| Song 2019 | English | Prevalence | OS | 52 | 4931497 | Others | Others | conventional MA |
| Song 2020 | English | Intervention | Both | 5 | 930 | Therapeutic clinical intervention | Biophysical status | conventional MA |
| Song 2020 | English | Intervention | RCT | 3 | 203 | Therapeutic clinical intervention | Morbidity | conventional MA |
| Suh 2020 | English | Diagnosis | RCT | 16 | 4080 | Diagnostic test | Morbidity | no MA conducted |
| Suh 2020 | English | Prognosis | RCT | 23 | 8387 | Diagnostic test | Others | conventional MA |
| Sun 2021 | English | Intervention | OS | 5 | 597047 | Health behavior | Morbidity | conventional MA |
| Tang 2020 | English | Prevalence | OS | 55 | 6327 | Others | Morbidity | conventional MA |
| Torres-Aguila 2019 | English | Prognosis | OS | 61 | 88807 | Biological status | Morbidity | no MA conducted |
| Trimboli 2018 | English | Diagnosis | Both | 7 | 1187 | Diagnostic test | Morbidity | conventional MA |
| Tsivgoulis 2019 | English | Prognosis | Both | 28 | 4531 | Therapeutic clinical intervention | Morbidity | conventional MA |
| Vu 2021 | English | Prognosis | OS | 22 | 94575 | Biological status | Morbidity | conventional MA |
| Wang 2018 | English | Intervention | Both | 8 | 1502 | Therapeutic clinical intervention | Mortality | conventional MA |
| Wang 2020 | English | Intervention | RCT | 4 | 1024 | Therapeutic clinical intervention | Morbidity | conventional MA |
| Wang 2021 | English | Intervention | RCT | 8 | 1488 | Therapeutic clinical intervention | Symptoms | conventional MA |
| Yang 2020 | Korean | Other | OS | 24 | 26 | Health behavior | Morbidity | no MA conducted |
| Yang 2020 | English | Intervention | Both | 11 | 922 | Therapeutic clinical intervention | Others | conventional MA |
| Yoon 2018 | English | Intervention | Both | 11 | 395 | Therapeutic clinical intervention | Biophysical status | conventional MA |
| Yoon 2019 | English | Intervention | OS | 11 | 1407 | Therapeutic clinical intervention | Biophysical status | conventional MA |
| Yoon 2020 | English | Other | Both | 33 | NR | Others | Others | no MA conducted |
| Yoon 2020 | English | Intervention | Both | 57 | NR | Therapeutic clinical intervention | Morbidity | no MA conducted |
| Yoon 2021 | English | Diagnosis | OS | 14 | 1233 | Diagnostic test | Biophysical status | conventional MA |
| Yoon 2021 | English | Prognosis | OS | 7 | 241 | Biological status | Symptoms | conventional MA |
| Yu 2018 | Korean | Other | OS | 49 | 13194 | Others | Others | conventional MA |
| Yu 2020 | English | Prevalence | OS | 13 | 4041 | Biological status | Morbidity | conventional MA |
| Zhang 2018 | English | Intervention | RCT | 11 | 1289 | Therapeutic clinical intervention | Symptoms | conventional MA |
| Zhang 2019 | English | Prognosis | OS | 17 | 13468 | Others | Morbidity | conventional MA |
| Zhong 2021 | English | Intervention | RCT | 93 | 504613 | Therapeutic clinical intervention | Morbidity | network MA |

MA, meta-analysis; OS, observational studies; RCT, randomized controlled trials
